# Rifampicin resistance in mycobacterium tuberculosis patients using GeneXpert at Livingstone Central Hospital for the year 2015: a cross sectional explorative study

**DOI:** 10.1186/s12879-017-2750-9

**Published:** 2017-09-22

**Authors:** Sepiso K. Masenga, Harrison Mubila, Benson M. Hamooya

**Affiliations:** 1 University of Zambia, School of Health Sciences, P.o Box 50110, Lusaka, Zambia; 2 University of Zambia, School of Public Health, P.o Box 50110, Lusaka, Zambia; 3 Pathology Laboratory department, Research section, Livingstone Central Hospital, P.o Box 60091, Livingstone, Zambia; 4 Chikankata College of Biomedical Sciences, Southern province, Zambia

**Keywords:** Rifampicin resistance, Mycobacterium tuberculosis, GeneXpert

## Abstract

**Background:**

Since the recent introduction of GeneXepert for the detection of Tuberculosis (TB) drug resistance mutations in both primary resistance and acquired resistance in Zambia, little has been documented in literature on the issue of rifampicin resistance especially in the face of a high National TB burden. The study aimed to determine the prevalence of rifampicin resistance in tuberculosis patients at Livingstone Central Hospital for the year 2015.

**Methods:**

This was a cross sectional study conducted at Livingstone Central Hospital where we reviewed 152 records (from January 1, 2015 to 31st December, 2015) involving patients who presented with clinically suspected TB or documented TB, whose samples were sent to the laboratory for GeneXpert Mycobacterium tuberculosis/rifampicin testing. Statistical evaluations used a one-sample test of proportion and Fisher’s exact test.

**Results:**

The age of participants ranged from 8 months to 73 years old (median = 34). Of the participants with complete data on gender, 99 (66%) and 52 (34%) were males and females respectively. The TB co-infection with HIV prevalence was 98.3% (*p* < 0.001). Prevalence of rifampicin resistance was 5.9% and there was no statistical significant difference between being male or female (*p* = 0.721).

**Conclusion:**

We were able to show from our study, evidence of rifampicin resistance at Livingstone Central Hospital. Hence, there was need for further in-depth research and appropriate interventions (i.e close follow-up and patient care for drug resistance positive patients).

## Background

Tuberculosis (TB) burden in Zambia is high and continues to pose a serious public health challenge [1] in spite of the implementation of the Directly Observed Treatment Short-course (DOTS) strategy as recommended by the World Health Organization (WHO) [2].

Molecular assays such as GeneXpert are changing the landscape of the diagnosis and management of drug resistant TB and may prove to be a cost-effective solution to this problem in a variety of settings [3]. GeneXpert uses real-time polymerase chain reaction (PCR) to detect the specific sequence for Mycobacterium tuberculosis (MTB) as well as that of rifampicin resistance [3]. The detection of rifampicin resistance serves as a surrogate marker for detecting Multi-drug Resistant Tuberculosis (MDR-TB), resistance to at least isoniazid and rifampicin [4]. Rifampicin resistance mechanism involves missense mutations in the rifampin resistance-determining region (RRDR) of the rpoB gene and 95% of strains that are resistant to rifampicin, harbor a mutation within the 81-bp region of the rpoB gene [5]. Reports indicate that in Africa the prevalence of resistance to one or more anti-TB drugs and of MDR-TB range from 3% to 37.3% and 1.4% to 11.6%, respectively [6–9]. Furthermore, the rapid spread of MDR-TB especially in new TB patients is challenging the effectiveness of TB control programmes in many low income countries, including Zambia [10].

Drug resistance results from genetic mutation in specific genes and delay in the recognition of drug resistance results in a delay in initiation of treatment or effective therapy, which is the major factor that contributes to MDR-TB outbreaks [5].

Our preliminary findings at Livingstone Central Hospital demonstrated that analysis of 3675 specimens by smear microscopy and GeneXpert confirmed only 177 cases of TB, giving a prevalence of 4.8%. This number could even be higher in reality owing to the missing information in certain cases. Of all the 2374 cases examined by GeneXpert, only about 157 (6.6%) had TB. We therefore, conducted an exploratory study that was to provide baseline data and reveal the magnitude of rifampicin resistance at Livingstone Central Hospital, as well as help in planning, management and care of TB patients. The data generated would serve as basis for future interventional studies.

This study was also important owing to paucity of studies in Zambia reporting on prevalence of rifampicin resistance as there were only 21 sites performing GeneXpert Mycobacterium tuberculosis/rifampicin testing (MTB/RIF) by end of 2014. The aim of our study was to determine the prevalence of rifampicin resistance in TB patients at Livingstone Central Hospital.

## Methods

### Study area and population

The study was conducted at Livingstone Central Hospital (LCH), Livingstone, Zambia. Livingstone Central Hospital (LCH) is a referral hospital for specialized conditions within Livingstone and other areas of the province. The TB resistance testing in the district is only done at LCH and most TB patients are also HIV positive. The estimated number of HIV positive people on antiretroviral therapy (ART) in Livingstone district was 62,317. At LCH alone, as of September, 2013, the estimated number of HIV positive adults enrolled at the ART Clinic was 6601, of which 3356 (Males = 1423, Females = 1933) were currently on ART**.**

### Study design and data collection

This was a cross-sectional study which reviewed all records involving patients who presented with clinically suspected TB whose samples were referred to the laboratory for GeneXpert MTB/RIF for diagnosis. GeneXpert MTB/RIF positives were repeated.

Eligibility criteria used: We included in the study, all individuals with a positive test for tuberculosis and all that had GeneXpert rifampicin resistance results in the year 2015. Unfortunately, drug resistance results for the samples showing rifampicin resistance positive, was not available to ascertain MDR-TB or rifampicin mono-resistance.

We employed standard Laboratory protocols based on published guidelines [11] .

For data analysis, STATA version 12 was used to generate all the tables shown in the results section. Statistical evaluations used a one-sample test of proportion (to determine the statistical significance of two proportions arising from the same sample, with the hypothesis that the proportions were equal) and Fisher’s exact test for expected value/s of less than five in any cell of a 2 × 2 table. Significance level was at *p*-value of 0.05, below which, statistical significance was accepted.

## Results

### Basic characteristics of study participants

The study consisted of 152 participants, of which most of them were males (99; 65.6%). The difference was statistically significant (*p* < 0.001). Only 151 had complete information on gender and 120 on age. The median age among the study participants was 34 years (range: 0.8–73).

### Clinical characteristics of the study participants

The majority in the study population were HIV positive (118/120; 98.3%) as compared to those without HIV and the difference was statistically significant (*p* < 0.0001). HIV status for 32 patients was not recorded. Among the study participants, the prevalence of detected rifampicin resistance was 5.9% (9/152) (p < 0.0001).

As shown in Table 1 below, there was no statistical difference between males (55.6%) and females (44.4%) with respect to rifampicin resistance, *p* = 0.4989. Of the nine patients with rifampicin resistant TB, 7 were HIV positive; the remaining 2 patients had no recorded HIV status.. There was no significant difference in rifampicin resistance among MTB grading (*p* = 0.912).

**Table 1:** Comparison between those with or without Rifampicin and demographic & characteristics

characteristic	n (%)		*p*-value
	RR detected	RR Not detected	
Sex^a^			
Male	5 (55.6)	89 (65.0)	0.721^f^
Female	4 (44.4)	48 (35.0)	0.4989^prr^
Total	9 (100)	137 (100)	
HIV status^b^			
HIV Negative	0 (0)	2 (1.8)	1.000^f^
HIV Positive	7 (100)	110 (98.2)	
Total	7 (100)	112 (100)	
Mycobacteria Tuberculosis grading (MTB)^c^			
Very low	1 (25.0)	29 (22.0)	0.912^f^
Low	1 (25.0)	52 (39.4)	
Medium	1 (25.0)	28 (21.2)	
High	1 (25.0)	23 (17.4)	
Total	4 (100)	132 (100)	

*RR* Rifampicin resistance, ^prr^one sample test of proportions between males and females who had RR, ^f^ Fisher’s exact test used

^a^sex was not recorded for 6 patients hence not factored in the statistical test

^b^HIV status was not recorded for 33 patients

^c^Mycobacteria Tuberculosis grading was not recorded for 16 patients

## Discussion

### Rifampicin resistance prevalence by gender

In this study we showed that GeneXpert identified 9 (5.9%) cases of rifampicin resistant TB out of the total (152) cases of TB in the year 2015 at Livingstone Central Hospital (LCH). The prevalence of rifampicin resistance in the current study is consistent with other published studies [12, 13]. However, a much lower and higher proportion was found in a study which was conducted in India [14] and in Ghana [3], respectively. The discrepancies maybe attributable to differences in sample sizes as well as poor record keeping and capturing of data alluded to in their studies as well as many other factors that may be unknown to us.

In this study, it was revealed that gender was not a factor when it comes to rifampicin resistance among our study population. This is similar to a study which was conducted in India [14], where it was found that the risk of rifampicin resistance among genders was the same. This could have been so due to the fact that men and women are exposed equally to factors that lead to rifampicin resistance in our settings, such as adherence and HIV. However, the low numbers of patients with rifampin resistance in this study may limit the comparison between males and females. Furthermore, we found no significant relationship between rifampicin resistance and all the demographic variables considered in this study. The male preponderance trend in TB in contrast to females suggests that there are more males than females who get diagnosed with TB. This trend is similar to other studies conducted in Nigeria where there were more (84, 60%) male patients against 56 (40%) females [15], in India [14] and in Zambia [1] where there were more male cases than female cases (*P* = 0.035). Further, this is very consistent with global trends in TB by gender [16].

### Rifampicin resistance with HIV co-infected participants

In this study HIV co-infection was not found to be statistically significant with anti-TB drug resistance, as found in other studies [17] [18] however, the prevalence of co-infection with HIV among TB patients was very evident (98.3%, *p* < 0.001). This trend is very consistent with previous reports where two-thirds of Zambian TB patients notified to the NTP were co-infected with HIV [10]. The case of MDR-TB and co-infection with HIV may need further investigation as the cases of HIV negative participants were very few (2) in the sample population under our study. A study conducted in Zambia [13] that reviewed records from 2000 to 2011 found no data available on MDR-TB / HIV co-infections and this was attributed to inadequate reporting and recording.

On clinical basis, drug resistance is divided into two types namely: primary resistance and acquired resistance. Primary resistance occurs in individuals who have never been treated for TB and are infected with a resistant MTB strain. On the other hand, acquired resistance develops during therapy for TB. Though, it was beyond the scope of our study to ascertain the causes of drug resistance, it is well known from research in literature that TB resistance to drugs is multifactorial in nature and investigations into these factors must be specific to get a clear insight [19, 20].

### Limitations

There was significant missing information on certain variables such as age, HIV status as well as rifampicin resistance status on patient records. Missing variables that are critical to finding the underlying causes of rifampicin resistance was a real limitation to our study. In this study we did not have contact with the patients hence certain important information such drug adherence, type of medications etc. which are important factors in the genesis of drug resistance, were not available for collection.

In this study, no information was available on drug resistance testing for the patients that tested rifampicin resistance positive on GeneXpert MTB/RIF to ascertain whether they were MDR-TB cases or rifampicin mono-resistant cases. This lack of information is very common in Zambian laboratories with few exceptions, such as the reference laboratories at the University Teaching Hospital, Tropical Disease Research Centre and the Chest Diseases Laboratory (National TB Reference Laboratory) [13]. Thus, it is possible that previous studies from this country under-estimated the prevalence of MDR-TB [13] and indicate that further investigations are warranted and illustrate the importance of improving the documentation of MDR-TB in Zambia.

## Conclusions

The study was able to reveal rifampicin resistance at Livingstone Central Hospital. There is need for further in-depth research and appropriate interventions such as close follow-up and patient care for drug resistant patients.

## Abbreviations

ART: antiretroviral therapy
DOTS: Directly observed treatment short-course
LCH: Livingstone Central Hospital
MDR-TB: Multi-drug resistance Tuberculosis
MTB/RIF: Mycobacterium tuberculosis/rifampicin testing
MTB: Mycobacterium Tuberculosis
RR: Rifampicin resistance
RRDR: rifampin resistance-determining region
TB: Tuberculosis
WHO: World Health Organization
XDR-TB: Extensively Drug Resistant TB
